# Safety of meglumine gadoterate (Gd-DOTA)-enhanced MRI compared to unenhanced MRI in patients with chronic kidney disease (RESCUE study)

**DOI:** 10.1007/s00330-012-2705-x

**Published:** 2012-12-05

**Authors:** Gilbert Deray, Olivier Rouviere, Lorenzo Bacigalupo, Bart Maes, Thierry Hannedouche, François Vrtovsnik, Claire Rigothier, Jean-Marie Billiouw, Paolo Campioni, Joaquin Ferreiros, Daniel Devos, Daniel Alison, François Glowacki, Jean-Jacques Boffa, Luis Marti-Bonmati

**Affiliations:** 1 Department of Nephrology, Pitié Salpétrière Hospital, Bat G. Cordier, 47-83 Bd de l’hôpital, 75651 Paris cedex 13, France; 2 Hospices Civils de Lyon, Department of Urinary and Vascular Imaging, Hôpital E. Herriot, Université de Lyon, Lyon, France; 3 Université Lyon 1, faculté de médecine Lyon Est, Lyon, France; 4 Université Lyon 1, faculté de médecine Lyon Est, Lyon, France; 5 Radiology Department, E.O. Ospedali Galliera, Genova, Italy; 6 Department of Nephrology, Heilig Hartziekenhuis Roeselare, Roeselare, Belgium; 7 Department of Nephrology, University Hospitals, Strasbourg, France; 8 Department of Nephrology, Bichat Hospital, Paris, France; 9 Department of Nephrology Transplantation Dialysis, Pellegrin Hospital, Bordeaux, France; 10 Department of Nephrology, Onze Lieve Vrouw Ziekenhuis, Aalst, Belgium; 11 Azienda Ospedaliero-Universitaria Sant’Anna, Ferrara, Italy; 12 Servicio de Radiodiagnostico, Hospital Clinico de San Carlos, Madrid, Spain; 13 Department of Radiology, Gent University Hospital, Gent, Belgium; 14 Department of Radiology, Trousseau Hospital, Tours, France; 15 Department of Nephrology, University Hospitals, Lille, France; 16 Department of Nephrology and Dialysis, Tenon Hospital, Paris, France; 17 Department of Radiology, University of Valencia, Valencia, Spain

**Keywords:** Gadolinium-based contrast agent-induced nephropathy, Gd-DOTA, Meglumine gadoterate, MRI, Nephrogenic systemic fibrosis

## Abstract

**Objective:**

To prospectively compare the renal safety of meglumine gadoterate (Gd-DOTA)-enhanced magnetic resonance imaging (MRI) to a control group (unenhanced MRI) in high-risk patients.

**Methods:**

Patients with chronic kidney disease (CKD) scheduled for MRI procedures were screened. The primary endpoint was the percentage of patients with an elevation of serum creatinine levels, measured 72 ± 24 h after the MRI procedure, by at least 25 % or 44.2 μmol/l (0.5 mg/dl) from baseline. A non-inferiority margin of the between-group difference was set at −15 % for statistical analysis of the primary endpoint. Main secondary endpoints were the variation in serum creatinine and eGFR values between baseline and 72 ± 24 h after MRI and the percentage of patients with a decrease in eGFR of at least 25 % from baseline. Patients were screened for signs of nephrogenic systemic fibrosis (NSF) at 3-month follow-up.

**Results:**

Among the 114 evaluable patients, one (1.4 %) in the Gd-DOTA-MRI group and none in the control group met the criteria of the primary endpoint [Δ = −1.4 %, 95%CI = (−7.9 %; 6.7 %)]. Non-inferiority was therefore demonstrated (*P* = 0.001). No clinically significant differences were observed between groups for the secondary endpoints. No serious safety events (including NSF) were noted.

**Conclusion:**

Meglumine gadoterate did not affect renal function and was a safe contrast agent in patients with CKD.

***Key points*:**

*• Contrast-induced nephropathy (CIN) is a potential problem following gadolinium administration for MRI.*

*• Meglumine gadoterate (Gd-DOTA) appears safe, even in patients with chronic kidney disease.*

*• Gd-DOTA only caused a temporary creatinine level increase in 1/70 such patients.*

*• No case or sign of NSF was detected at 3-month follow-up.*

## Introduction

Contrast-induced nephropathy (CIN), part of a broader spectrum of acute kidney injuries [1], was first reported after administration of iodinated contrast media (CM) [2]. The European Society of Urogenital Radiology (ESUR) guidelines define CIN as acute kidney injury within days following CM administration when alternative causes of renal damage have been excluded [1]. The development of CIN after administration of gadolinium-based contrast agents (GBCAs) remains controversial, especially in high-risk groups such as patients with chronic kidney disease (CKD), in whom CIN has been inconsistently reported after injection of various GBCAs [3–5]. These discrepancies could be due to multiple factors: different doses and routes of administration of GBCAs, heterogeneous study designs and CIN definitions with the use of different formulas to estimate the glomerular filtration rate (eGFR), non-systematic use of prophylactic measures, and possible differences between GBCAs in terms of their intrinsic nephrotoxic potential [4]. CIN has never been described after administration of meglumine gadoterate (Gd-DOTA) alone, a molecule in which gadolinium is chelated (“caged”) by a macrocyclic ligand. The absence of CIN after Gd-DOTA administration was initially demonstrated in a small, randomised study conducted in CKD patients [6]. The absence of impact of Gd-DOTA on renal function was further documented in a large Japanese post-marketing study [7], and its good overall safety was confirmed in a surveillance study of 84,621 patients, including 764 patients with renal failure [8]. In a retrospective study conducted by Ergün et al [9] in 91 patients with stage 3 and 4 CKD [mean eGFR by the modified MDRD formula (Modification of Diet in Renal Disease): 33 ml/min/1.73 m^2^] assessed by magnetic resonance imaging (MRI) with an intravenous dose of 0.2 mmol/kg of Gd-DOTA, gadopentetate dimeglumine, or gadodiamide, 11 patients (12.1 %) developed CIN. However, the respective CIN rate for each GBCA was not specified, and differences in renal toxicity between these GBCAs therefore cannot be excluded. The primary objective of this prospective study was to assess the CIN rate in a similar population of patients with stable stage 3 and 4 CKD undergoing Gd-DOTA-enhanced MRI compared with a control group undergoing unenhanced MRI.

## Subjects and methods

### Study design

This phase IV (RESCUE trial), open-label, non-randomised, multinational study compared the renal safety of Gd-DOTA-enhanced MRI with unenhanced MRI in high-risk patients. The study was registered at ClinicalTrials.gov, no. NCT00650845. Institutional Review Board and regulatory approval was granted for each centre and all patients gave their written informed consent.

### Patients

Patients (male or female, aged ≥18 years) with known stable stage 3 or 4 CKD according to the Kidney Disease Improving Global Outcomes (KDIGO) definition (i.e. eGFR >15 ml/min/1.73 m^2^ and <60 ml/min/1.73 m^2^) scheduled to undergo MRI were included.

Patients were ineligible when surgery or chemotherapy was planned within 72 h post-procedure; if they had undergone an imaging procedure (MRI or computed tomography with or without contrast medium) during the 7 days before inclusion, or within 72 h post-procedure; if they had participated in any investigational drug study during the 30 days prior to inclusion or were scheduled to participate in another study within 72 h post-procedure; or had a known allergy to GBCAs.

Patients with a contraindication to MRI, a diagnosis of haemodynamic instability or acute myocardial infarction during the 2 weeks prior to inclusion, requiring haemodialysis, with newly diagnosed unstable diabetes, or pregnant women were ineligible. All patients who had received any medication known to be nephrotoxic or cause elevation of serum creatinine levels during the 2 weeks before the baseline blood sample and throughout the study duration were not included in the study (a list of nephrotoxic medications was established at the beginning of the trial and given to each site). Moreover, at the end of the study, all patient medications were reviewed by an independent expert (nephrologist) blinded to the imaging procedure performed. Any included patient having received nephrotoxic treatment was identified and considered as a protocol deviation.

A patient was considered to be screened but not yet included until the first blood sample had been drawn within 1 day before MRI (baseline). A patient was definitively included in the absence of any exclusion criteria and when (1) the relative difference between baseline serum creatinine and a previous serum creatinine value, obtained at least 1 week and less than 6 months before the baseline blood test, did not exceed 15 % and (2) the eGFR value according to the abridged MDRD study prediction equation [10] was >15 ml/min/1.73 m^2^ and <60 ml/min/1.73 m^2^.

### MRI procedure and Gd-DOTA administration

According to the investigator’s judgement (i.e. diagnosis needed) and the hospital’s standard practices, patients were assigned to the Gd-DOTA-enhanced MRI group or the unenhanced MRI group. In each centre, MRI procedures were performed according to the hospital’s standard protocols. Gd-DOTA (Dotarem®, Guerbet, Roissy CdG, France) was to be injected intravenously by a power injector at a dose of 0.1 mmol/kg (0.2 ml/kg).

### Safety assessment

Serum creatinine and other laboratory parameters (sodium, potassium, bicarbonate, calcium, uric acid, haematocrit, and haemoglobin) were assayed in the same laboratory for each patient for both pre- and post-MRI blood samples.

The primary endpoint was nephrotoxicity, defined as the percentage of patients with serum creatinine level elevation, determined 72 ± 24 h after MRI, of at least 25 % or 44.2 μmol/l (0.5 mg/dl) above the baseline value.

Secondary endpoints were: variation in (1) serum creatinine and (2) eGFR values between baseline and 72 ± 24 h after MRI as well as the percentage of patients with a decrease in eGFR of at least 25 % from baseline values, the percentage of patients who met the criteria of the primary endpoint and for whom serum creatinine returned to the baseline value 14 days after MRI, and the potential influence on renal function of the measures to prevent CIN (medication/hydration). There was no specific hydration protocol defined in this study and the sites followed their routine practice. Nonetheless, whenever possible, the patient was encouraged to drink liberally before and after the injection. Special attention was paid to acute and delayed allergy-like reactions. The number of dialysis sessions after MRI was also recorded.

Patients were monitored for adverse events from the time informed consent was signed until 72 ± 24 h after MRI. In case of CIN, renal function was to be checked via a third blood sample 14 days after the imaging procedure in the same laboratory as the two others. The patient was then followed up until complete resolution of the CIN.

Adverse events reported by the local investigators were classified as serious or non-serious and assessed according to their clinical severity (mild, moderate, or severe) and their relationship (possible, doubtful, or not related) to the study contrast agent or the unenhanced-MRI procedure. Outcomes of adverse events were classified into the following categories: resolved with or without sequelae, ongoing, worsened at the time of the report, or death.

Vital signs (blood pressure, pulse) were monitored just before each MRI procedure, then 15 min and 1 h after the procedure.

Each patient was contacted 3 months after the MRI exam to detect any symptoms or signs suggestive of nephrogenic systemic fibrosis (NSF). This 3-month follow-up was chosen according to the average time of NSF onset as suggested by several publications [11–14].

### Statistical analysis

All statistical analyses were performed with SAS version 9.2 software (SAS Institute Inc, Cary, NC) at the *P* < 0.05 level of significance.

Based on data published by Ergün et al. [9], we assumed an average incidence of 12 % CIN in each group. A total of 120 evaluable patients was considered to be a sufficient sample size to ensure, with 80 % power and at a 5 % one-sided significance level, that non-inferiority of Gd-DOTA-MRI over unenhanced MRI could be demonstrated with a fixed −15 % non-inferiority margin. In other words, non-inferiority would be demonstrated if the lower bound of the 95 % confidence interval (CI) of the difference of percentage of patients who met the criteria of the primary endpoint (unenhanced-MRI patients – Gd-DOTA MRI patients) was greater than −15 %. Assuming a 10 % dropout rate during the study, a total of 134 patients were deemed necessary to achieve the study objectives. The secondary endpoints (variation from baseline of serum creatinine concentration and eGFR level, or decrease in eGFR level from baseline ≥25 %) were investigated by regression models with adjustment for centres.

Student’s *t* test and Fisher’s exact test were used for the other parameters.

Two data analysis populations were defined from all patients screened in the study.

The Evaluable Safety Population (ESP) included all patients for whom two blood samples were available for pre- and post-MRI serum creatinine assay. This population was used for analysis of the primary endpoint and all other safety analyses (including adverse events, laboratory data, and vital signs).

The per-protocol (PP) population was a subpopulation of ESP, i.e. evaluable patients not presenting any significant protocol deviations or violations. This population was used for analysis of the primary endpoint as well as serum creatinine and eGFR level variations from baseline.

Propensity score analysis was planned to balance the absence of randomisation to assign patients to unenhanced or Gd-DOTA-enhanced MRI except when baseline statistical tests demonstrated that the two groups were homogeneous at inclusion.

## Results

### Patients eligible for analysis

**Fig. 1:**
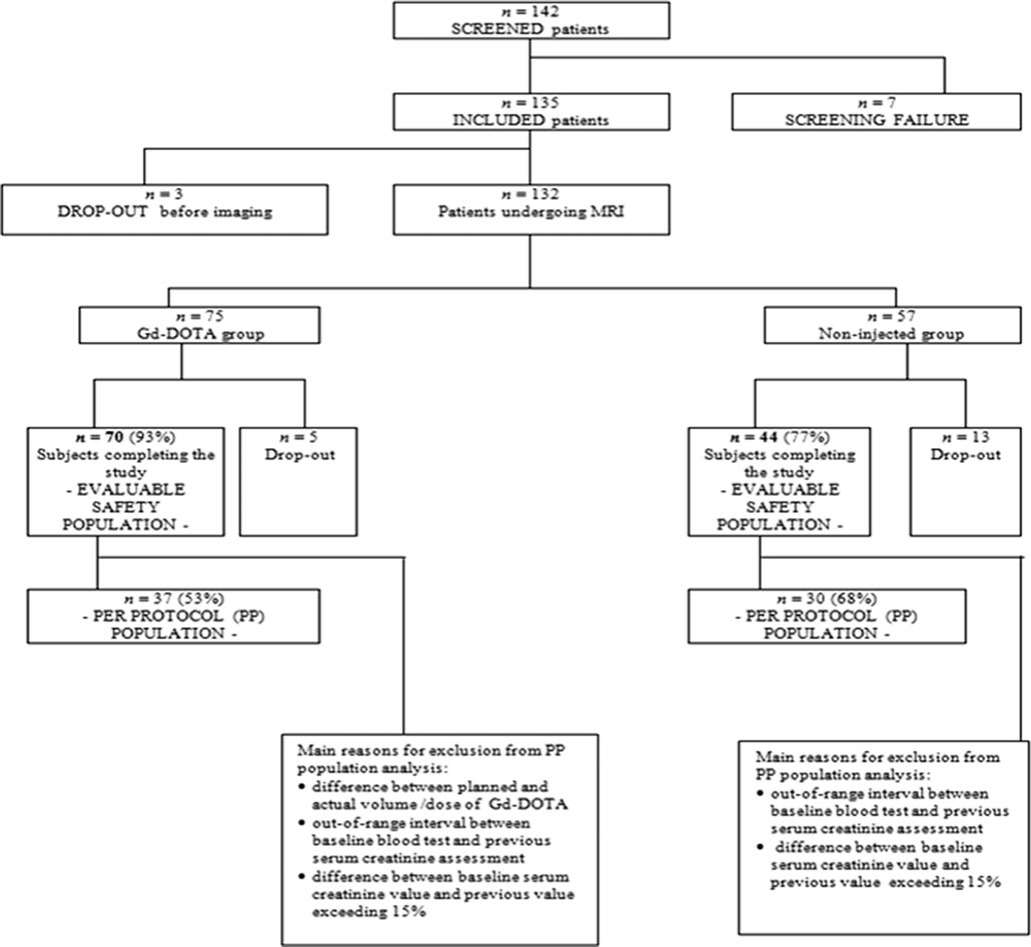
Study patient flow chart

A total of 142 patients were screened in 15 centres in Europe (Belgium, France, Italy, and Spain), 135 were included, and 114 were evaluable for the primary endpoint (70 in the Gd-DOTA MRI group and 44 in the unenhanced-MRI group). Patient disposition is described in the study patient flow chart (Fig. 1).

As shown in Table 1, the two groups of the ESP were well balanced in terms of demographic and baseline data. No significant difference was observed between the two groups for sex ratio (*P* = 0.529), age (*P* = 0.428), body mass index (*P* = 0.790), baseline serum creatinine (*P* = 0.367) level, or baseline eGFR level (*P* = 0.641).

**Table 1:** Demographic and baseline characteristics, evaluable safety population

Baseline characteristics	Gd-DOTA-MRI (*n* = 70)	Unenhanced MRI (*n* = 44)	Total (*n* = 114)	P-values^a^
Age (years)	69.1 ± 11.5	67.3 ± 12.0	68.4 ± 11.7	*P* = 0.428
Mean ± SD (min/max)	(34/92)	(26/86)	(26/92)	
Gender *n* (%)				*P* = 0.529
Male	47 (67.1 %)	27 (61.4 %)	74 (64.9 %)	
Female	23 (32.9 %)	17 (38.6 %)	40 (35.1 %)	
BMI (kg/m^2^)	27.5 ± 4.9^b^	27.3 ± 3.9	27.4 ± 4.6	*P* = 0.790
Mean ± SD (min/max)	(16.0/41.2)	(18.8/36.4)	(16.0/41.2)	
Serum creatinine (μmol/l)	175.9 ± 65.4	165.3 ± 59.2	171.5 ± 62.8	*P* = 0.367
Mean ± SD (min/max)	(79.6/371.3)	(88.4/344.8)	(79.6/371.3)	
eGFR (ml/min/1.73 m^2^)	37.58 ± 13.6	38.78 ± 12.6	38.04 ± 13.2	*P* = 0.641
Mean ± SD (min/max)	(15.0/82.0)	(17.0/65.3)	(15.0/82.0)	
History of allergy, *n* (%)	12 (17.1 %)	7 (15.9 %)	19 (16.7 %)	*P* = 1.000
Premedication/prehydration, *n* (%)	2 (2.9 %)	0 (0.0 %)	2 (1.8 %)	*P* = 0.517

^a^Student’s *t*-test, chi-square, Fisher’s exact test

^b^Mean BMI calculated on 68 reported values (2 missing)

A history of known allergic reactions was reported in 12 patients (17.1 %) in the Gd-DOTA-MRI group and 7 patients (15.9 %) in the unenhanced-MRI group.

The various types of MRI procedures are shown in Table 2. The most common procedures were body MRI (43.9 %) and MR angiography (21.9 %). No significant between-group differences were observed.

**Table 2:** Indications for Gd-DOTA-MRI and unenhanced-MRI examinations, evaluable safety population

MRI study area (*n*,%)	Gd-DOTA-MRI^a^ (*n* = 70)	Unenhanced MRI^a^ (*n* = 44)	Total^a^ (*n* = 114)	P-values (Fisher’s exact test)
Body (abdomen/thorax)	37 (52.9 %)	13 (29.5 %)	50 (43.9 %)	
Kidney	23 (32.9 %)	10 (22.7 %)	33 (28.9 %)	*P* = 0.134
Pelvis	8 (11.4 %)	1 (2.3 %)	9 (7.9 %)	
Limbs	4 (5.7 %)	0 (0.0 %)	4 (3.5 %)	
Liver	0 (0.0 %)	2 (4.5 %)	2 (1.8 %)	
Pancreas	1 (1.4 %)	0 (0.0 %)	1 (0.9 %)	
Heart	1 (1.4 %)	0 (0.0 %)	1 (0.9 %)	
Angiography	20 (28.6 %)	5 (11.4 %)	25 (21.9 %)	
Renal	15 (21.4 %)	4 (9.1 %)	19 (16.7 %)	*P* = 0.781
Carotid	1 (1.4 %)	1 (2.3 %)	2 (1.8 %)	
Aorto-iliac	2 (2.9 %)	0 (0.0 %)	2 (1.8 %)	
Other	2 (2.9 %)	0 (0.0 %)	2 (1.8 %)	
Musculoskeletal system (bones/joints)	2 (2.9 %)	14 (31.8 %)	16 (14.0 %)	–
Central nervous system	1 (1.4 %)	9 (20.5 %)	10 (8.8 %)	
Brain	1 (1.4 %)	7 (15.9 %)	8 (7.0 %)	*P* = 1.000
Head/neck	0 (0.0 %)	2 (4.5 %)	2 (1.8 %)	
Other indications	10 (14.3 %)	6 (13.6 %)	16 (14.0 %)	–

^a^A patient with multiple indications is counted several times

Gd-DOTA was administered intravenously at a mean dose of 0.1 mmol/kg (range: 0.06–0.29 mmol/kg) and at a mean flow rate of 2 ml/s (range: 0.7–4 ml/s).

### Primary endpoint: serum creatinine level increase from baseline ≥25 % or ≥44.2 μmol/l (0.5 mg/dl)

As shown in Table 3, in the ESP, one patient (1.4 %) in the Gd-DOTA-MRI group and no patients in the control group met the criteria of the primary endpoint with a mean difference (unenhanced-MRI – Gd-DOTA-MRI) of −1.4 % [95 % CI = (−7.9 %; 6.7 %)]. As the lower bound of the 95 % CI (−7.9 %) was superior to the non-inferiority margin (−15 %), the non-inferiority of Gd-DOTA-MRI over unenhanced MRI was demonstrated (*P* = 0.001). Consistent results were observed in the PP population (67 patients), with a mean difference of −2.7 % [95%CI = (−14.1 %; 8.9 %), *P* = 0.0204].

**Table 3:** Primary endpoint: number (%) of patients with serum creatinine level variation from baseline ≥25 % or ≥44.2 μmol/l (0.5 mg/dl) in the evaluable safety population and per-protocol population

	Gd-DOTA-MRI	Unenhanced MRI	Test
Evaluable safety population	(*n* = 70)	(*n* = 44)	Difference (unenhanced-MRI – Gd-DOTA-MRI) = −1.4 %
	1 (1.4 %)	0 (0.0 %)	Exact 95 %CI = [−7.9 %; 6.7 %]
			*P* = 0.001^a^
Per-protocol population	(*n* = 37)	(*n* = 30)	Difference (unenhanced-MRI – Gd-DOTA-MRI) = −2.7 %
	1 (2.7 %)	0 (0.0 %)	Exact 95 %CI = [−14.1 %; 8.9 %]
			*P* = 0.0204^b^

^a^P-value testing the difference −1.4 % vs. −15 % (non-central Student’s *t*-test)

^b^P-value testing the difference −2.7 % vs. −15 % (non-central Student’s *t*-test)

In the patient who met the criteria of the primary endpoint (male subject, age: 68 years with type 2 diabetes mellitus, body mass index: 33.4 kg/m^2^; Gd-DOTA dose: 0.095 mmol/kg), the baseline serum creatinine level (176.8 μmol/l (2 mg/dl)) rose to 229.8 μmol/l (2.6 mg/dl) after MRI, i.e. a relative increase of 30 % and an absolute increase of 53 μmol/l (0.6 mg/dl), and returned to baseline within 2 weeks.

### Secondary endpoints

As shown in Table 4, no significant difference in serum creatinine level variation from baseline was observed in the ESP (−1.40 % in the Gd-DOTA group vs. –3.48 % in the unenhanced group). This was confirmed in the PP population (0.05 % in the Gd-DOTA group vs. –5.17 % in the unenhanced group).

**Table 4:** Serum creatinine and eGFR level variations from baseline, evaluable safety population and per-protocol population

Secondary endpoints	Gd-DOTA-MRI	Unenhanced MRI	Student’s *t*-test
Serum creatinine level variation from baseline (%) (mean ± SD, min/max)			
Evaluable safety population	(*n* = 70)	(*n* = 44)	Difference (Gd-DOTA-MRI - unenhanced-MRI) = 2.08 %
	−1.40 ± 10.36	−3.48 ± 9.92	95 %CI = [−1.80 %; 5.97 %]
	(−25.00/30.00)	(−28.57/18.05)	*P* = 0.291
Per-protocol population	(*n* = 37)	(*n* = 30)	Difference (Gd-DOTA-MRI - unenhanced MRI) = 5.21 %
	0.05 ± 10.84	−5.17 ± 9.16	95 %CI = [0.24 %; 10.18 %]
	(−21.02/30.00)	(−28.57/14.46)	*P* = 0.040
eGFR level variation from baseline (%) (mean ± SD, min/max)			
Evaluable safety population	(*n* = 70)	(*n* = 44)	Difference (Gd-DOTA-MRI - unenhanced-MRI) = −2.53 %
	3.02 ± 12.51	5.55 ± 12.94	95 %CI = [−7.37 %; 2.30 %]
	(−26.12/39.37)	(−17.42/47.45)	*P* = 0.301
Per-protocol population	(*n* = 37)	(*n* = 30)	Difference (Gd-DOTA-MRI - unenhanced-MRI) = −6.22 %
	1.37 ± 12.65	7.58 ± 12.82	95 %CI = [−12.46 %; 0.03 %]
	(−26.12/31.30)	(−14.43/47.45)	*P* = 0.051

The same trend is observed for the eGFR level variation from baseline in the ESP (3.02 % in the Gd-DOTA group vs. 5.55 % in the unenhanced group) and PP (1.37 % in the Gd-DOTA group vs. 7.58 % in the unenhanced group). A 26 % decrease in the eGFR value was observed in the single patient with CIN (baseline eGFR: 35.4 ml/min/1.73 m^2^ and post-MRI eGFR: 26.2 ml/min/1.73 m^2^).

As shown in Table 1, the rate of use of patient hydration or other prophylactic measures was very low in the two groups (2 patients in the Gd-DOTA-MRI group and no patients in the unenhanced-MRI group).

In both groups, no patients developed acute and delayed allergy-like reactions, and no patients required dialysis after MRI.

No adverse events were observed in the unenhanced-MRI group. Five adverse events were observed in five patients (7.1 %) after Gd-DOTA-enhanced MRI (abdominal pain, haematoma, constipation, toothache, and increase of blood creatinine levels). One adverse event (hypotension) occurred before Gd-DOTA injection. No statistically significant between-group difference was observed (*P* = 0.080). All adverse events were non-serious, mild, resolved within 3 weeks, and were not related to Gd-DOTA injection (except for elevation of serum creatinine levels in the single patient with CIN, which was possibly related to Gd-DOTA).

Vital sign variations from baseline (Table 5) were comparable between groups (except for systolic blood pressure at the 1-h time point), and no clinically significant out-of-range variation was observed. Variations from baseline in haematological and biochemistry parameters were comparable between groups (Table 5). All values out of the normal range were classified as not clinically significant.

**Table 5:** Vital signs and laboratory data variations from baseline, evaluable safety population

Vital signs	Gd-DOTA-MRI (*n* = 70)	Unenhanced MRI (*n* = 44)	Student’s *t*-test
Diastolic blood pressure (mmHg) (mean ± SD, min/max)			
15 min	(*n* = 64)	(*n* = 41)	*P* = 0.427
	2.84 ± 10.73	1.15 ± 10.48	
	(−20/31)	(−21/35)	
1 h	(*n* = 60)	(*n* = 40)	*P* = 0.173
	0.62 ± 11.58	−2.25 ± 7.76	
	(−20/42)	(−19/19)	
Systolic blood pressure (mmHg) (mean ± SD, min/max)			
15 min	(*n* = 64)	(*n* = 41)	*P* = 0.067
	4.84 ± 16.52	−1.29 ± 16.58	
	(−30/50)	(−39/40)	
1 h	(*n* = 60)	(*n* = 40)	*P* = 0.044
	1.62 ± 17.86	−5.70 ± 17.14	
	(−36/60)	(−47/35)	
Heart rate (bpm) (mean ± SD, min/max)			
15 min	(*n* = 63)	(*n* = 40)	*P* = 0.501
	0.62 ± 12.15	2.00 ± 5.47	
	(−56/34)	(−11/12)	
1 h	(*n* = 59)	(*n* = 40)	*P* = 0.568
	0.90 ± 9.66	1.95 ± 7.80	
	(−22/35)	(−15/16)	
Laboratory data	Gd-DOTA-MRI (*n* = 70)	Unenhanced MRI (*n* = 44)	Student’s *t*-test
Laboratory data – relative variations from baseline values (mean ± SD, min/max)			
Bicarbonate	(*n* = 65)	(*n* = 30)	*P* = 0.423
	−0.01 ± 0.09	0.01 ± 0.11	
	(−0.22/0.23)	(−0.23/0.35)	
Calcium	(*n* = 64)	(*n* = 40)	*P* = 0.825
	−0.01 ± 0.04	−0.00 ± 0.04	
	(−0.13/0.15)	(−0.14/0.09)	
Haematocrit	(*n* = 67)	(*n* = 40)	*P* = 0.364
	−0.02 ± 0.05	−0.01 ± 0.07	
	(−0.17/0.12)	(−0.17/0.24)	
Haemoglobin	(*n* = 67)	(*n* = 41)	*P* = 0.345
	−0.02 ± 0.04	−0.01 ± 0.07	
	(−0.16/0.10)	(−0.16/0.30)	
Potassium	(*n* = 69)	(*n* = 43)	*P* = 0.426
	−0.01 ± 0.08	−0.02 ± 0.09	
	(−0.20/0.23)	(−0.24/0.19)	
Sodium	(*n* = 70)	(*n* = 43)	*P* = 0.646
	0.00 ± 0.02	−0.00 ± 0.01	
	(−0.04/0.04)	(−0.05/0.02)	
Uric acid	(*n* = 61)	(*n* = 41)	*P* = 0.197
	−0.01 ± 0.08	−0.03 ± 0.11	
	(−0.16/0.17)	(−0.29/0.26)	

No signs suggestive of NSF were observed at 3-month follow-up.

As baseline statistical tests showed that the two groups were well balanced in terms of demographic and baseline data, the results of the propensity score analysis did not provide any additional value.

## Discussion

This prospective study showed a very similar low rate of CIN after Gd-DOTA-enhanced MRI (1.4 %) and unenhanced MRI (0 %) in patients with stage 3 or 4 CKD. Apart from two small trials conducted with GBCAs versus a control group [6, 15], RESCUE is the only large-scale prospective study in CKD patients comparing GBCA-enhanced MRI to unenhanced MRI. This is an important point, as the serum creatinine level has been reported to increase in patients not receiving contrast material just as frequently as in series of patients who received contrast material [16].

The primary endpoint strictly complied with the current ESUR guidelines [1]. Despite similar patient characteristics, the same intravenous route of administration and a less stringent definition of CIN than that reported by Ergün et al.—elevation of serum creatinine levels by at least 44.2 μmol/l (0.5 mg/dl) over baseline values within 24–72 h after CM administration [9]—our 1.4 % CIN rate was lower than the 12.1 % rate reported in the previously published retrospective study, which was used to define the −15 % non-inferiority margin. The higher dose of GBCA used by Ergün et al. (0.2 mmol/kg) might be an explanation. Although the −15 % margin is now questionable in view of the low CIN rate observed in our prospective study, it is to be noted that, according to protocol hypothesis, the 120 subjects of RESCUE would have allowed demonstrating non-inferiority with a margin set as low as -4.5 %, so these results show that patients undergoing Gd-DOTA-enhanced MRI did not have any clinically significant increased risk of CIN.

In the other four studies [17–20] in which GBCA-related nephrotoxicity was reported in CKD patients, two studies [17, 18] also reported a higher CIN rate (50 % and 28 %, respectively). Compared to RESCUE, CIN occurred in populations with similar degrees of CKD, but with globally smaller number of patients (10 [17] and 25 [18]), definitions of CIN that did not strictly comply with ESUR guidelines—more than 50 % decrease in GFR [17], at least 44.2 μmol/l (0.5 mg/dl) increase of baseline serum creatinine levels at 48 h, or need for dialysis within 5 days [18]—and high doses of CM: 0.57 ± 0.17 mmol/kg [17] or 0.6 ± 0.3 mmol/kg [18] for angiographic examinations. The common denominator of the three studies [9, 17, 18] was the use of GBCAs with different structures: gadopentetate dimeglumine [9], gadodiamide [9, 18], gadobutrol [17, 18], and Gd-DOTA [9]. This could at least partly explain the higher CIN rates compared to that observed with Gd-DOTA alone, although a number of published studies have also shown that administration of these GBCAs is associated with no or a low rate of CIN in CKD patients [3–5] and have even reported a serum creatinine level decrease after the administration of gadolinium [21]. In the remaining two studies [19, 20], conducted with some of these GBCAs, lower CIN rates were observed: 1.9 % [19] and 2.5 % [20]. The differences with RESCUE, despite higher doses of GBCAs, were either a more stringent definition of CIN (at least 1.0 mg/dl increase in baseline serum creatinine levels at 48 h and oligoanuria [19]) in a similar CKD population (mean baseline creatinine clearance: 38.2 ± 16 ml/min) [19] or a less severe CKD population (46.8 % of patients with mild CKD) with a different CIN definition (greater than 25 % decrease of baseline GFR) [20].

The mechanism of the putative nephrotoxicity of GBCAs is unknown (related to the different GBCA structures or free gadolinium) [3, 4]. High doses of GBCAs have been recognised as a risk factor in CKD patients [1], but even approved doses have been shown to be associated with occasional cases of CIN [22]. Acute tubular necrosis lesions, following sequential administration of two GBCAs, have been described in the only case documented by kidney biopsy [23].

Another possible explanation for the low CIN rate observed in our study would be biases induced by methodological issues. The heterogeneous indications for MRI did not allow randomisation of procedures (Gd-DOTA-enhanced MRI versus unenhanced MRI) or a double-blind design, as the investigators determined the type of procedure according to the patient’s condition and underlying diseases. However, despite the open-label design and absence of randomisation, the baseline demographic characteristics, especially mean serum creatinine and mean eGFR levels, were comparable between the two groups. The greater number of patients in the Gd-DOTA group excluded from the PP population did not affect the results of the primary endpoint in the PP analysis compared to the ESP analysis.

Finally, measures to prevent CIN would not provide an explanation, as they were rarely used.

The results of this study confirm the absence of impact of Gd-DOTA on renal function, as already observed when administered to a small cohort of ten patients with CKD undergoing MRI compared to ten patients not receiving CM [6]. The mean (standard error of the mean) serum creatinine values (μmol/l) at baseline and 48 h post-procedure were 330.9 (42.2) and 306.2 (44.7), respectively, in the Gd-DOTA group and 329.7 (53.9) and 344.6 (64.9), respectively, in the control group. This absence of impact on renal function was also recently documented in a post-marketing study conducted in 3,444 Japanese patients [7]. Kidney function tests, performed as part of routine medical care in patients with impaired renal function, did not reveal any significant difference in terms of serum creatinine values (μmol/l; mean ± standard deviation) before (194.5 ± 194.5) and after (203.3 ± 194.5) Gd-DOTA administration.

Nephrogenic systemic fibrosis is a rare but occasionally life-threatening complication of GBCA exposure that occurs in patients with severe CKD [4, 24]. No unconfounded case of NSF has so far been reported with Gd-DOTA administration, and no signs of this condition were detected at 3-month follow-up in the present study. This is in accordance with a statement recently issued by the European Medicines Agency [25] where macrocyclic chelates including Gd-DOTA were considered to be low-risk for NSF.

In conclusion, no clinically relevant differences in terms of the risk of nephrotoxicity (CIN) and general safety profile were observed in a population of high-risk patients with stable stage 3 and 4 CKD undergoing MRI after intravenous injection of Gd-DOTA compared to those not receiving Gd-DOTA.
